# Responses of mature symbiotic nodules to the whole-plant systemic nitrogen signaling

**DOI:** 10.1093/jxb/eraa221

**Published:** 2020-05-09

**Authors:** Ilana Lambert, Marjorie Pervent, Antoine Le Queré, Gilles Clément, Marc Tauzin, Dany Severac, Claire Benezech, Pascal Tillard, Marie-Laure Martin-Magniette, Stefano Colella, Marc Lepetit

**Affiliations:** 1 Laboratoire de Symbioses Tropicales et Méditerranéennes, INRAE, IRD, CIRAD, SupAgro, Univ. Montpellier, Montpellier, France; 2 Institut Jean-Pierre Bourgin, INRAE, AgroParisTech, CNRS, Université Paris-Saclay, Versailles, France; 3 MGX, CNRS, INSERM, Univ. Montpellier, Montpellier, France; 4 Biologie et Physiologie Moléculaire des Plantes, INRAE, CNRS, SupAgro, Univ. Montpellier, Montpellier, France; 5 Institute of Plant Sciences Paris-Saclay (IPS2), Université Paris-Saclay, Univ. Evry, CNRS, INRAE, Orsay, France; 6 Institute of Plant Sciences Paris-Saclay (IPS2), Université de Paris, CNRS, INRAE, Orsay, France; 7 UMR MIA-Paris, AgroParisTech, INRAE, Université Paris-Saclay, Paris, France; 8 University of Warwick, UK

**Keywords:** Legumes, mature nodules, nitrogen, *Rhizobium*, sugar partitioning, symbiosis, systemic signaling

## Abstract

In symbiotic root nodules of legumes, terminally differentiated rhizobia fix atmospheric N_2_ producing an NH_4_^+^ influx that is assimilated by the plant. The plant, in return, provides photosynthates that fuel the symbiotic nitrogen acquisition. Mechanisms responsible for the adjustment of the symbiotic capacity to the plant N demand remain poorly understood. We have investigated the role of systemic signaling of whole-plant N demand on the mature N_2_-fixing nodules of the model symbiotic association *Medicago truncatula*/*Sinorhizobium* using split-root systems. The whole-plant N-satiety signaling rapidly triggers reductions of both N_2_ fixation and allocation of sugars to the nodule. These responses are associated with the induction of nodule senescence and the activation of plant defenses against microbes, as well as variations in sugars transport and nodule metabolism. The whole-plant N-deficit responses mirror these changes: a rapid increase of sucrose allocation in response to N-deficit is associated with a stimulation of nodule functioning and development resulting in nodule expansion in the long term. Physiological, transcriptomic, and metabolomic data together provide evidence for strong integration of symbiotic nodules into whole-plant nitrogen demand by systemic signaling and suggest roles for sugar allocation and hormones in the signaling mechanisms.

## Teaser

The effect of systemic signaling of whole-plant N demand on mature N_2_-fixing nodules was investigated. Sugar allocation and hormones are candidates to integrate symbiotic nodules to whole-plant nutritional demand.

## Introduction

A hallmark trait of legumes is to form nodules with soil bacteria called rhizobia. The symbiotic root organs allow the plant to acquire nitrogen (N) from the air. In nodules, terminally differentiated bacteroids fix N_2_ and supply NH_4_^+^ to the plant, while sucrose, synthesized in the leaves by the plant and exported to the nodules by the phloem, is the source of carbon (C) and energy fueling symbiotic N_2_ fixation (SNF). NH_4_^+^ produced by nitrogenase is exported from the bacteroids into the cytosol of the infected cell, where it is rapidly assimilated by GS/GOGAT (glutamine synthetase/glutamate synthase) in the presence of α-ketoglutarate to synthesize amino acids that are exported outside of the nodule (mainly asparagine in the case of *Medicago truncatula*). Efflux transporters of the SWEET family are likely to be involved in the translocation of sucrose from the phloem to the nodule (Kryvoruchko *et al.*, 2016; Sugiyama *et al.*, 2017). Once unloaded in the nodule, sucrose is generally metabolized by sucrose synthase (Baier *et al.*, 2007). UDP-glucose and free hexoses are produced, which, after phosphorylation by hexokinases, enter the glycolytic or oxidative pentose phosphate pathways and are metabolized to malate that is finally imported into the bacteroid to fuel the tricarboxylic acid (TCA) cycle (Udvardi and Day, 1997). In bacteroids, the anaplerotic ‘γ-aminobutyric acid (GABA) shunt’ involving pyruvate and GABA (most probably provided by the plant) has been proposed to bypass two steps of the TCA cycle by producing succinate semialdehyde, alanine, and finally succinate (Prell *et al.*, 2009). This pathway might enhance energy generation under hypoxic conditions and, therefore, might improve the efficiency of SNF (Prell *et al.*, 2009; Sulieman and Schulze, 2010; Sulieman, 2011).

The carbon metabolite cost of SNF is elevated, and the plant generally favors mineral N nutrition when the mineral N resource is not limiting plant growth. Indeed, formation of symbiotic organs requires low mineral N availability, whereas high levels of the mineral N repress SNF, inhibit nodule formation, and trigger nodule senescence (Streeter and Wong, 1988). Cysteine protease genes up-regulated in response to high nitrate concentrations are responsible for bacteroid proteolysis (Pérez Guerra *et al.*, 2010; Pierre *et al.*, 2014). Although air is an unlimited N source (air is composed of 80% N_2_), plant symbiotic N acquisition is frequently spatially and/or temporally limited because nodules are highly sensitive to local environmental constraints, such as drought, that strongly inhibit SNF (Durand *et al.*, 1987; Serraj *et al.*, 1999; Gil-Quintana *et al.*, 2013). Symbiotic organs are controlled by the local N environment of the root, but also by systemic signals originating from the other organs to adjust symbiotic capacities to the N demand and the photosynthetic capacity of the whole plant. Split-root systems involving N_2_-fixing *Medicago*/*Sinorhizobium* holobionts allowed characterization of these controls. The availability of a high level of mineral N was associated with the systemic repression of SNF (Ruffel *et al.*, 2008). Plant N limitation by suppressing SNF of one side of a split-root system (Ar/O_2_ treatment) was associated with systemic stimulation of mature nodule growth and new nodule initiations on the other N_2_-fixing roots not directly exposed to the treatment (Jeudy *et al.*, 2010; Laguerre *et al.*, 2012). This stimulation was associated with an increased allocation of photosynthates to these roots (Jeudy *et al.*, 2010). Several longstanding hypotheses to explain the modulation of SNF by the plant have been proposed. A popular model is related to the so-called ‘nodule oxygen diffusion barrier’ that tightly correlates with the SNF activity of mature nodules (Sheehy *et al.*, 1983; Hunt and Layzell, 1993). However whether the variations of the oxygen flux inside the nodule are the cause or the consequence of the regulation of SNF remains unclear. Another hypothesis is an ‘N-feedback’ inhibition of SNF by downstream N metabolites accumulated in the plant, including glutamate, asparagine, or GABA (Parsons *et al.*, 1993; Bacanamwo and Harper, 1997; Sulieman *et al.*, 2010; Sulieman and Schulze, 2010; Sulieman, 2011). The amino acid auxotrophy of bacteroids would be consistent with such control (Lodwig *et al.*, 2003). Finally, as carbon metabolites provided by the plant to the nodule are the primary nutritional source of bacteroids, a control of SNF by sucrose synthase and organic acid allocation has also been suggested (González *et al.*, 1995; Schwember *et al.*, 2019). However, the molecular mechanisms involved in these controls remain unknown, and none of these models has yet been validated. Nodule formation is under the control of the N status of the plant and the systemic repression of pre-existing nodules by the mechanism of autoregulation of nodule number (AON; Kosslak and Bohlool, 1984; reviewed by Ferguson *et al.*, 2019). Super-/hypernodulating mutants impaired in AON form nodules in the presence of a high level of mineral N, suggesting a role for this pathway in the control of nodulation by N signaling (Olsson *et al.*, 1989). Nevertheless, because the systemic response of mature nodule growth to N demand remains active in such mutants, the systemic control of mature nodules is likely to be operated by an AON-independent pathway (Jeudy *et al.*, 2010). Another pathway, acting in parallel with AON, involved in the stimulation of nodule formation in the plant under mineral N deficit has been described but it has little (if any) impact on mature nodule development (Huault *et al.*, 2014; Laffont *et al.*, 2019).

The plant transcriptional reprogramming associated with nodule formation and functioning has been characterized (El Yahyaoui *et al.*, 2004; Mitra and Long, 2004; Lohar *et al.*, 2006; Benedito *et al.*, 2008; Libault *et al.*, 2010; Moreau *et al.*, 2011; Breakspear *et al.*, 2014; Roux *et al.*, 2014). It involves the up-regulation of hundreds of specific plant genes with roles in the early signaling responses, bacterial infection, nodule formation, bacteroid differentiation, and SNF activation (Oldroyd *et al.*, 2011). These two late phases are marked by the induction of large families of genes encoding nodule-specific cysteine-rich (NCR) and glycine-rich nodule-specific peptides (GRP) associated with bacteroid differentiation (Kereszt *et al.*, 2018) as well as the genes encoding leghemoglobins allowing the bacterial nitrogenase to be active (Rutten and Poole, 2019). Bacteroid differentiation is characterized by the up-regulation of bacterial genes involved in SNF under microoxic conditions, but also by the down-regulation of an extensive number of the genes expressed in free-living cells (Becker *et al.*, 2004; Capela *et al.*, 2006). A network of plant hormones tightly regulates the symbiotic developmental program. Ethylene has been implicated in the negative control of nodule formation and nodule infection (Guinel, 2015; Reid *et al.*, 2018). Modulating shoot methyl jasmonate or cytokinin (CK) accumulation has suggested roles for these molecules in the systemic control of nodulation in *Lotus japonicus* (Nakagawa and Kawaguchi, 2006; Kinkema and Gresshoff, 2008; Sasaki *et al.*, 2014; Azarakhsh *et al.*, 2018). CLE and CEP hormone peptides have been implicated in the long-distance control of nodule formation and AON as well as in root formation and nitrate acquisition (Mortier *et al*., 2012; Okamoto *et al.*, 2016; Taleski *et al.*, 2018; Laffont *et al.*, 2019). However, the role of these peptides in mature nodules remains poorly documented.

A few studies have shown that the addition of a high level of mineral N to the roots of the holobionts is associated with extensive nodule transcriptome reprogramming affecting metabolism and development (Moreau *et al.*, 2011; Cabeza *et al.*, 2014). However, mineral N is a local signal by itself that is known to strongly affect the root transcriptome (Li *et al.*, 2014). These studies did not discriminate between the local effects of mineral N (i.e. at the site of application) and the systemic effects (i.e. related to the satisfaction of the whole-plant N demand). A previous report based on the analysis of split-root systems has shown that whole-plant systemic N signaling has a substantial impact on the transcriptome of whole nodulated roots, but the effects on nodule formation and/or mature nodule development could not be separated (Ruffel *et al.*, 2008). In this study, we have investigated the impact of systemic N signaling on mature N_2_-fixing nodules of *M. truncatula*. We used split-root systems to investigate systemic N satiety and N deficit responses. We monitored N_2_ fixation, bacteroid senescence, metabolite accumulation, and plant and bacteroid transcriptomes in order to characterize the early responses.

## Materials and methods

### Split-root plant growth condition

Seeds of *M. truncatula* genotype A17 were scarified in 97% H_2_SO_4_ for 5 min and cold-treated at 4.0 °C in water for 48 h, before germination at room temperature in the dark. After 4 d, the primary root tips were cut to promote branching of the root system. Individual plantlets were transferred into hydroponic culture tanks containing a vigorously aerated basal nutrient solution renewed every week comprising 1 mM KH_2_PO_4_, 1 mM MgSO_4_, 0.25 mM CaCl_2_, 0.25 mM K_2_SO_4_, 50 μM KCl, 30 μM H_3_BO_3_, 5 μM MnSO_4_, 1 μM ZnSO_4_, 1 μM CuSO_4_, 0.7 μM (NH_4_)_6_Mo_7_O_24_, and 100 μM Na-Fe-EDTA supplemented with 1 mM KNO_3_. The pH was adjusted to 5.8 with KOH. Plants were grown under 16 h light/8 h dark cycles, 250 μmol s^−1^ m^−2^ photosynthetically active radiation light intensity, 22 °C/20 °C day/night temperature, and 70% relative humidity. The 3-week-old plants were transferred to a nutrient solution adjusted to pH 7 supplemented with 0.5 mM KNO_3_ containing the *Sinorhizobium medicae* md4 bacteria (10^7^ cfu ml^–1^). Nodules appeared after 4–6 d and were functional after 2 weeks. Nutrient solutions, renewed every week, were adjusted to pH 7 but not supplemented with mineral N. For split-root experiments, the root systems of 5-week-old plants were separated into two parts, each side being installed in a separate compartment. Differential N treatments were initiated that modify the N provision to one side of the root system while the other side remains supplied with aerated nutrient solution without mineral N. The N-satiety treatment (SN_2_) consists of supplying the roots with a nutrient solution supplemented with 10 mM NH_4_NO_3_. The N-limitation treatment (DN_2_) consists of removing N_2_ from the treated compartment by applying a continuous flow of 80% argon/20% O_2_. Nutrient solutions were changed daily after the initiation of treatments.

### MD4 genome sequencing and annotation

Bacterial genomic DNA was extracted using the standard Doe Joint Genone Institute procedure. Genomic libraries were constructed with the Nextera XT DNA Library Prep Kit (Illumina). Sequencing was performed on illumina HiSeq 2500. Velvet (Zerbino and Birney, 2008), SOAPdenovo, and SOAPGapCloser (Luo *et al.*, 2012) packages were used to assemble the genome. Non-redundant contigs were ordered using the MAUVE aligner program (Darling *et al.*, 2004) utilizing the *Ensifer medicae* WSM419 genome as a reference, uploaded into the MicroScope platform, and subjected to an automatic annotation pipeline (Vallenet *et al.*, 2017). The NCBI genome sequence accession code is PRJEB29797.

### RNA sequencing (RNAseq) analysis

**Fig. 1.**
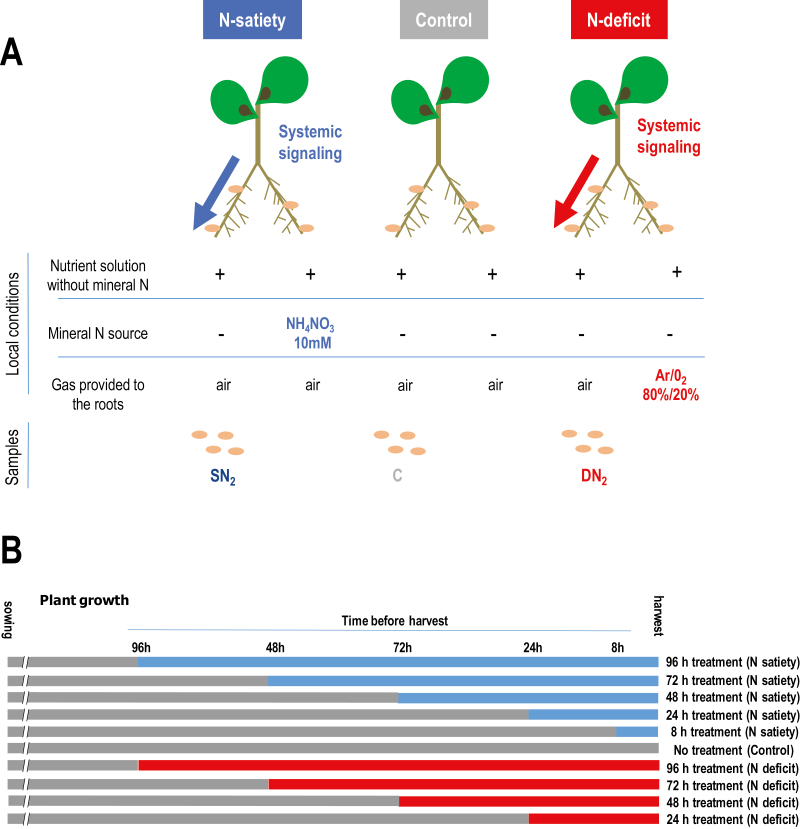
Split-root systems used to study N-deficit and N-satiety signaling on the mature nodules of symbiotic plants. Plants were grown hydroponically. (A) Treatments were applied on one half of root systems and effects were studied on the other sides on the SN_2_, C, DN_2_, nodules of N-satiety (blue), control (gray), and N-deficit (red) plants, respectively. N-satiety treatment (blue) was achieved by providing 10 mM NH_4_NO_3_. N-deficit treatment (red) was achieved by replacing air by Ar/O_2_ 80%/20% (v/v). Control plants (gray) were not treated. The untreated roots of all plants remained normally aerated with air. (B) N-satiety, N-deficit, and control plants of the same age were harvested simultaneously. Effect of the treatments (N-satiety and N-deficit) applied during different times before harvest were compared.

Nodule samples (150–200 mature nodules) were collected from split-root systems described in Fig. 1A. For each condition, triplicates were collected simultaneously and separately on three plants of the same age (Fig. 1B). Treatments consist of 0 (control), 1, or 3 d of N-satiety or N-deficit. RNAs were extracted from nodules using the miRNeasy® Mini Kit (Qiagen) according to the supplier’s recommendations. Plant and bacterial rRNAs were depleted using the strategy of Roux *et al.* (2014). Modified oligonucleotides used to capture rRNAs from *E. medicae* MD4 were described by Sallet *et al.* (2013) with the exception of 23S-1532-LNA that was replaced by AAG+TTAAG+CAAT+CCGTCACTA+CC (‘+’ preceding locked nucleotides) to match the sequence of *E. medicae* MD4. For each RNA extraction, polyadenylated plant mRNA- and rRNA-depleted total RNA libraries were generated and sequenced. They were prepared using the TruSeq Stranded mRNA Sample Preparation Kit (Illumina). Clustering and sequencing were performed in mode single-read 50 nt on illumina HiSeq 2500 according to the manufacturer’s instructions, consumables, and software. The sequencing reads (ArrayExpress database accession number E-MTAB-8597) were mapped on the *M. truncatula* v4.2 and the *E. medicae* MD4 strain genomes using the glint software (T. Faraut and E. Courcelle; http://lipm-bioinfo.toulouse.inra.fr). The edgeR (v.3.22.3) Bioconductor package (Robinson *et al.*, 2010) was used for differential expression analysis in R version 3.5.1. The counts per million method was used with a threshold of one read per million in half of the samples. Libraries were normalized using the Trimmed Mean of M-values method (Robinson *et al.*, 2010; Dillies *et al.*, 2013). The differential expression analysis was applied on DN_2_ and SN_2_ samples separately using generalized linear models (GLMs). For each treatment (N-deficit or N-satiety), the log of the average gene expression was used as an additive function of a replicate effect and a time effect (zero time is the no treatment control). A likelihood ratio test was performed to evaluate the expression change between two consecutive times, and the probabilities of significance were adjusted using the Benjamini–Hochberg procedure. Differentially expressed genes (DEGs) were selected using an adjusted *P*-value threshold of 0.05. The robustness of the analysis was confirmed by assaying the response to N signaling of nine DEG transcripts encoding leghemoglobin, cysteine protease, and SWEET11 in nodules collected from three independent split-root experiments (Supplementary Fig. S9 at *JXB* online). The co-expression analysis was performed by the ‘Coseq’ (v.1.4.0) Bioconductor R package (Rau and Maugis-Rabusseau, 2018). As clustering by the Coseq algorithm may vary depending on the initialization point, the clustering was repeated 40 times, and the integrated completed likelihood (ICL) was used as a criterion to choose the best modeling of the data. MtV4 annotation was supplemented with Plant metabolic network (PMN)-Mediccyc annotation of biochemical pathways (Urbanczyk-Wochniak and Sumner, 2007). Hypergeometric tests with a *P*-value threshold of 0.05 allowed for the identification of functional enrichment in particular gene subgroups as compared with their representation in the whole genome. Plant Gene Ontology (GO) analysis was performed with the online tools AgriGO (Tian *et al.*, 2017).

### Metabolomics and ^15^N_2_ fixation

Samples (pool of the mature nodules of two plants) were collected in five replicates after 0, 1, or 4 d of N-satiety or N-deficit treatments at the same time on plants of the same age. Metabolites were extracted and analyzed by GC/MS after grinding and homogenization in liquid nitrogen as described (Fiehn, 2006; Clément *et al.*, 2018). The ^15^N_2_ fixation activity was quantified according to Ruffel *et al.* (2008).

### Histological analyses

Longitudinal sections of fresh mature nodules (70 µm thickness) were transferred to the LIVE/DEAD® solution of the BacLight™ Bacterial Viability Kit for 15 min in darkness and then incubated in calco-fluorine solution (0.1 mg ml^–1^) for 15 min in the dark. The observations are made using a confocal fluorescence microscope (LSM 700, Zeiss).

## Results

### Split-root systems to characterize the responses of mature N_2_-fixing nodules to the whole-plant N demand

Split-root systems were used to investigate the systemic control exerted by the whole-plant N status on mature nodules. Roots of individual plants carrying mature N_2_-fixing nodules were separated in two compartments, and different N regimes were applied for 4 d (Fig. 1). All roots of control (C) plants were supplied with air and a nutrient solution without mineral N. ‘N-satiety’ plants grew in the same conditions, but a high level of mineral N (NH_4_NO_3_ 10 mM) was provided on half of their root system. ‘N-deficit’ plants had half of their roots supplied with a nutrient solution without mineral N, but aerated with a gas mixture of Ar/O_2_ 80%/20% instead of air (suppression of the N_2_ source), resulting in the immediate arrest of SNF at the site of treatment. Systemic effects of N treatment were investigated by comparing the SN_2_, DN_2_, and C nodulated roots (Fig. 1). SN_2_, DN_2_, and C nodules (Fig. 1) were in the same local condition, permissive to SNF (nutrient solution without mineral N and air), but connected to whole plants grown under ‘N-satiety’, ‘N-deficit’, and ‘control’ N regimes (resulting from the supply of the other side of their root system).

**Fig. 2.**
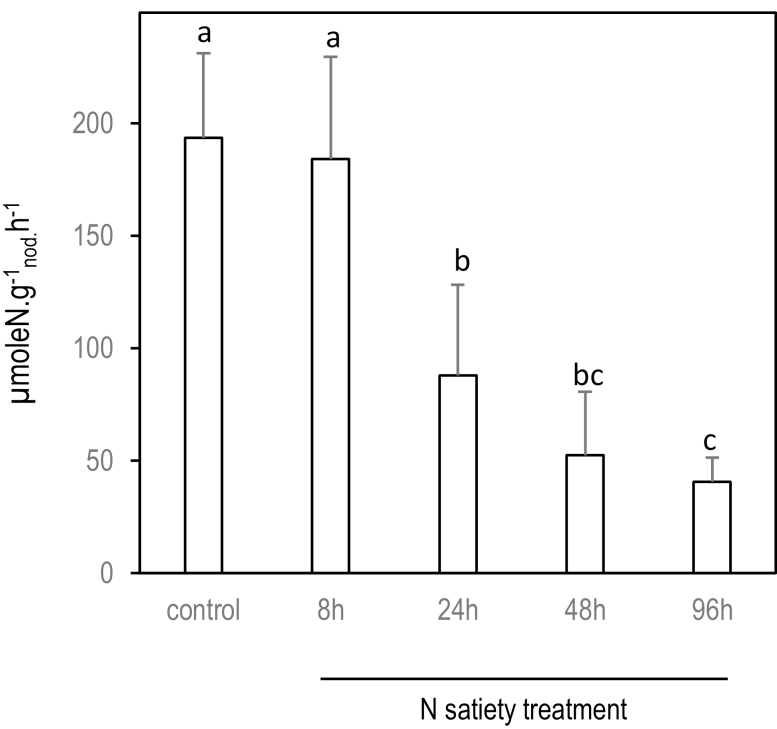
Effect of systemic N-satiety signaling on the specific SNF of SN_2_ mature nodules. N_2_ acquisition activity per biomass of nodule was measured using ^15^N_2_ labeling on the SN_2_ nodules exposed to 8, 24, 48, and 96 h of N-satiety treatment (the detailed experimental split-root design is described in Fig. 1). Measurements are made on 8–10 nodulated root samples collected from 8–10 split-root plants. Errors bars are the SD. Kruskal–Wallis tests (significance threshold of 0.05) followed by Wilcoxon pairwise comparisons between treatments with corrections for multiple testing (significance threshold of adjusted *P*-value of 0.05) were performed. Letters indicate distinct groups of values deduced from the Wilcoxon test.

**Fig. 3.**
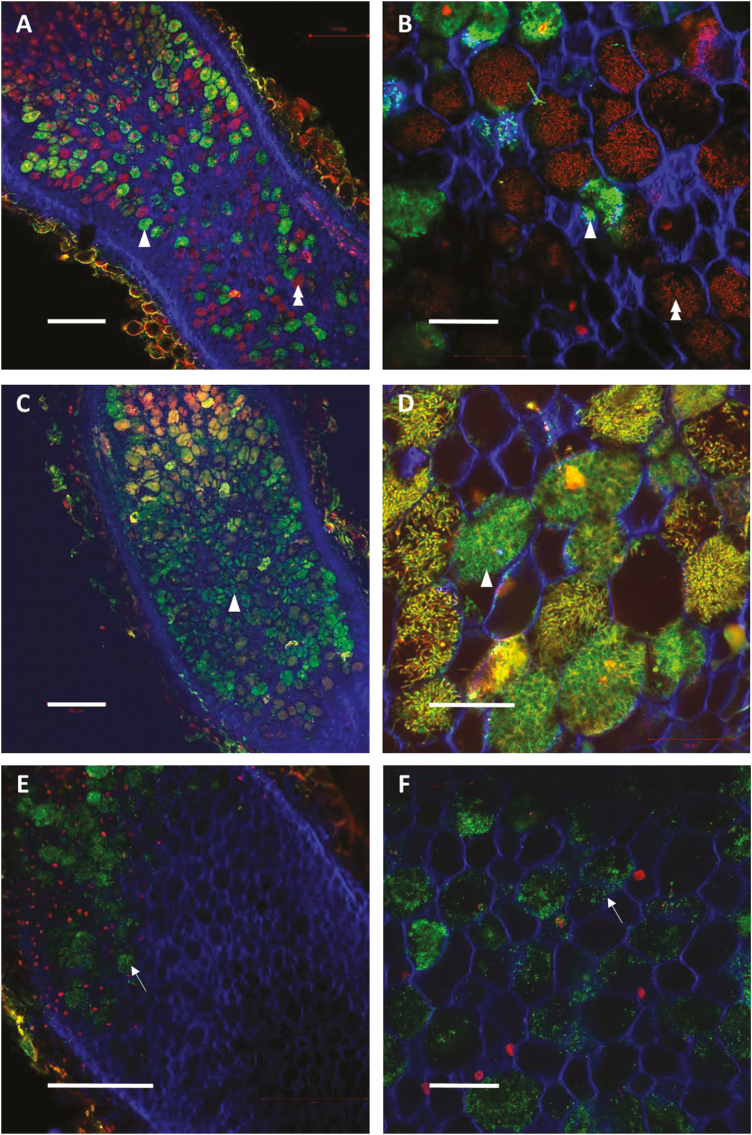
Effect of systemic N-satiety signaling on the bacteroid viability of C, DN_2_, and SN_2_ mature nodules. N-satiety split-root plants were cultivated as described in Fig. 1 and nodules were collected after 4 d of N-satiety treatment. Confocal microscopy images of transversal sections of control (A, B), DN_2_ (C, D), and SN_2_ (E, F) nodules upon live/dead and calco-fluorine staining. Viable bacteroids appear green (arrowheads) and dead bacteroids red (double arrowheads) inside plant cells. Control plants contained red- and green-labeled bacteroids. In SN_2_ nodules, bacteroids were almost completely destroyed, and only undifferentiated bacteria (arrows) were observable after 4 d (E, F). A higher level of green signal is found in DN_2_ nodules as compared with control nodules, suggesting delayed mortality compared with control nodules. Scale bars correspond to 200 µm in (A), (C), and (E), and 50 µm in higher magnification panels (B), (D), and (F).

Systemic repression of SNF by ‘N-satiety’ was monitored using ^15^N_2_ labeling after 8 h, and 1, 2, and 4 d of N treatment (Fig. 1B). We observed an extreme repression of the SNF of the SN_2_ nodules between 8 h and 24 h (Fig. 2) when compared with the control nodules (Fig. 3A, B). Nodule senescence was investigated by fluorescence imaging using life/dead staining in SN_2_ and DN_2_ nodules after 4 d of N treatments. Systemic ‘N-satiety’ signaling was associated with bacteroid senescence (Fig. 3E, F), whereas a reduction of bacteroid mortality was observed in response to systemic ‘N-deficit’ signaling (Fig. 3C, D).

To investigate the early metabolic and transcriptional responses to systemic N signaling potentially involved in the developmental responses (i.e. nodule senescence or nodule expansion) characterized after long-term treatments, we collected nodules during the first 4 d of treatments before significant differences in biomass allocation related to treatments could be measured.

### Root metabolite pool responses to systemic N signaling

**Fig. 4.**
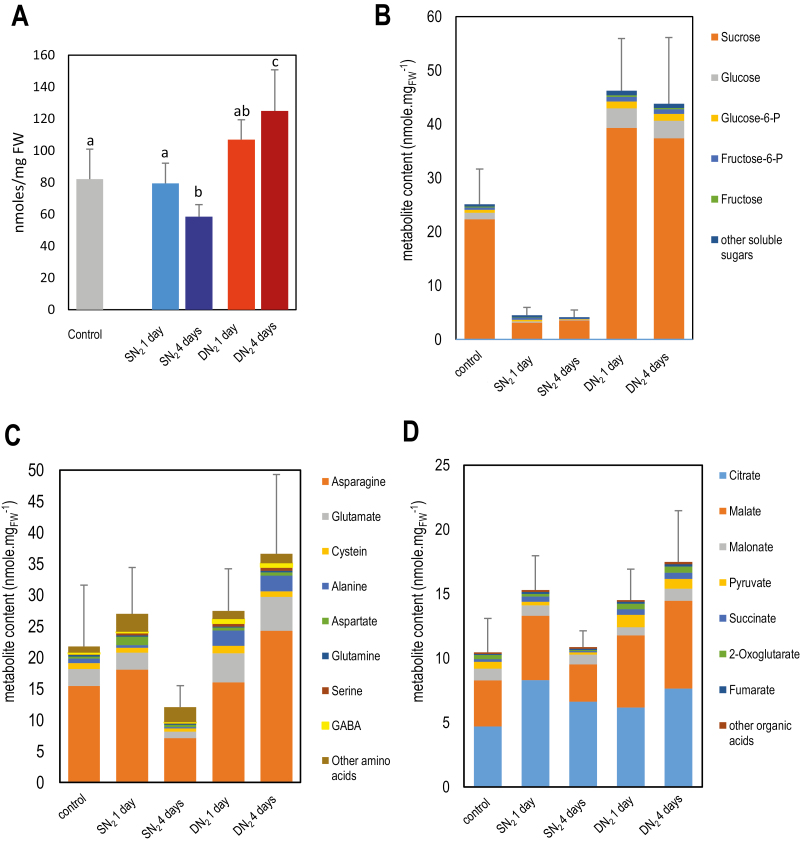
Effect of systemic N signaling on mature nodule metabolite content. Analysis was performed by GC/MS. Measurements were done on five independent biological replicates (one biological replicate is 200–300 nodules of two plants). Error bars are the SDs. (A) Total metabolite content. Gray, blue, and red bars correspond, respectively, to control, SN_2_, and DN_2_ nodules consistently with the colors in Fig. 1. We performed Kruskal–Wallis test (0.05 significance threshold) followed by Wilcoxon pairwise comparisons between treatments with corrections for multiple testing (0.05 significance threshold of the adjusted *P*-value). Letters indicate distinct groups of values deduced from the Wilcoxon test. (B) Sugar contents. Colors correspond to the five most abundant sugars of the nodules, representing >95% of the total sugar content of the mature nodules of control plants (complete data and statistical analysis are given in Supplementary Table S1). (C) Amino acid contents. Colors correspond to the eight most abundant amino acids of the nodules representing >95% of the total amino acid content of the mature nodules of control plants (complete data and statistical analysis are given in Supplementary Table S2) (D) Organic acid contents. Colors correspond to the seven most abundant organic acids of the nodules, representing >95% of the total organic acid content of mature nodules of control plants (complete data and statistical analysis are given in Supplementary Table S3).

Variation of soluble metabolite content associated with systemic N signaling was investigated by GC/MS analysis in C, SN_2_, and DN_2_ nodules after 1 d and 4 d of N treatments (Fig. 1). We detected 266 chemical species in the nodules, and we could unambiguously identify 107 of them. Most of them were soluble sugars, amino acids, and organic acids. Global soluble metabolite content varies in response to systemic N signaling: plant N-deficit stimulated their accumulation, whereas plant N-satiety decreased it (Fig. 4A).

Impacts of both N-deficit and N-satiety systemic signaling on the pool of soluble sugars in nodules were already observed after 1 d of treatment (Fig. 4B; Supplementary Table S1). N-satiety signaling was associated with a reduction of the content of sucrose, glucose, glucose 6-phosphate, fructose, and fructose 6-phosphate, and other minor sugars derived from them. Conversely, N-deficit stimulated the accumulation of these sugars in nodules. Nevertheless, levels of some minor sugars (e.g. galactose, xylose, and rhamnose) were affected to only a very small degree by the treatment, indicating that these extensive changes were not generalized to all sugars (Supplementary Table S1). Together with soluble sugar variations, we observed variations in nodule amino acid and organic acid contents (Fig. 4C, D; Supplementary Tables S2, S3). N-satiety signaling was associated with a transient stimulation of the accumulation of several of these metabolites after 1 d of treatment followed by a substantial reduction, while N-deficit stimulated their accumulation in mature nodules. Major amino acids such as asparagine, and glutamate, and minor ones such as alanine and GABA (Fig. 4C; Supplementary Table S2), displayed these variations, as did primary organic acids such as malate, pyruvate, succinate, and fumarate (Fig. 4D; Supplementary Table S3). Interestingly, succinic semialdehyde, a minor organic acid that is barely detectable in control samples and N-sufficient plants, accumulated in nodules under N-deficit (Fig. 4D; Supplementary Table S3). Together with GABA and alanine (Fig. 4C; Supplementary Table S2), they mark the stimulation of the ‘GABA shunt’ of the TCA cycle in response to systemic N-deficit signaling under the anaerobic conditions of mature nodules. Nonetheless, not all amino and organic acids followed these trends. Many minor nodule amino acids, such as tryptophan, valine, arginine, and isoleucine, were strongly accumulated in response to plant N-satiety and displayed only little variation under N-deficit (Fig. 4C; Supplementary Table S2). Systemic N signaling does not significantly affect citrate (the primary organic acid of the nodule) and malonate pools (Fig. 4D; Supplementary Tables S3). Altogether, these results revealed a specific impact of systemic N signaling on metabolite contents in nodules.

### Global responses of the mature nodule transcriptome to systemic N signaling

The effect of systemic N signaling on the transcriptome responses of the two symbiotic partners was investigated. We used the split-root systems described in Fig. 1A. DN_2_ or SN_2_ nodules were collected in triplicate on N-deficit or N-satiety plants (1 d or 3 d treatments) and compared with the nodules collected from control plants (Fig. 1B). To simultaneously monitor plant and bacterial gene expression, RNAseq analysis was performed on both polyadenylated RNA- and rRNA-depleted total RNAs. A total of 9110 plant DEGs in at least one condition (adjusted *P*-value <0.05) were identified (Supplementary Fig. S1; Supplementary Tables S4, S5). Systemic N signaling has a major impact on the nodule transcriptome as 44% of the plant genes expressed in the symbiotic organ are DEGs (39% and 13% in response to N-satiety and N-deficit, respectively). Only 5% of these genes were identified by the previous studies describing N responses of the symbiotic organs (Supplementary Fig. S2; Ruffel *et al.*, 2008; Cabeza *et al.*, 2014). Although N-deficit and N-satiety signaling targeted rather specific DEG groups, they also targeted a broad set of 1778 common genes (common DEGs) responding to both treatments (Supplementary Fig. S1). Total DEGs were strongly enriched with plant genes displaying responses to both treatments as compared with total expressed genes (hypergeometric test, *P*<0.01). N-satiety- and N-deficit-responsive genes display specific enrichments of some GO terms as compared with the entire transcriptome (Supplementary Table S6). Comparing these enrichments confirmed the specificities of the two systemic N responses (i.e. GO terms enriched in one group and not in the other), but also revealed enrichments of the same functions (i.e. the same GO terms enriched in both groups). Such massive transcriptome reprogramming was not observed in bacteria as only 674 differentially accumulated bacterial transcripts were identified (Supplementary Tables S7, S8), representing 11% and 1% of the expressed bacterial genes in response to N-satiety and N-deficit, respectively. Within the few N-deficit-responsive transcripts of the bacteria, we found no clear evidence of the transcriptional activation of the ‘GABA shunt’ of the TCA cycle at the level of metabolite accumulation. Most of the bacterial DEGs were identified in the nodules exposed to 3 d of N-satiety treatment (Supplementary Fig. S1B). However, at this stage of the treatment, the viability of bacteroids, the main fraction of bacterial cells present in the mature nodules, was probably impaired (Fig. 3). Bacteroid death had most probably (i) reduced the overall expression level of bacterial genes (that is assumed to be stable during the treatments); and (ii) modified the relative level of viable free-living versus differentiated bacteria in the samples. Therefore, the effect of long-term N-satiety treatments on the bacterial transcriptome needs to be interpreted with caution as the changes were probably related to bacteroid death and only indirectly to N signaling.

### Analysis of plant transcriptome reprogramming in response to systemic N signaling

**Fig. 5.**
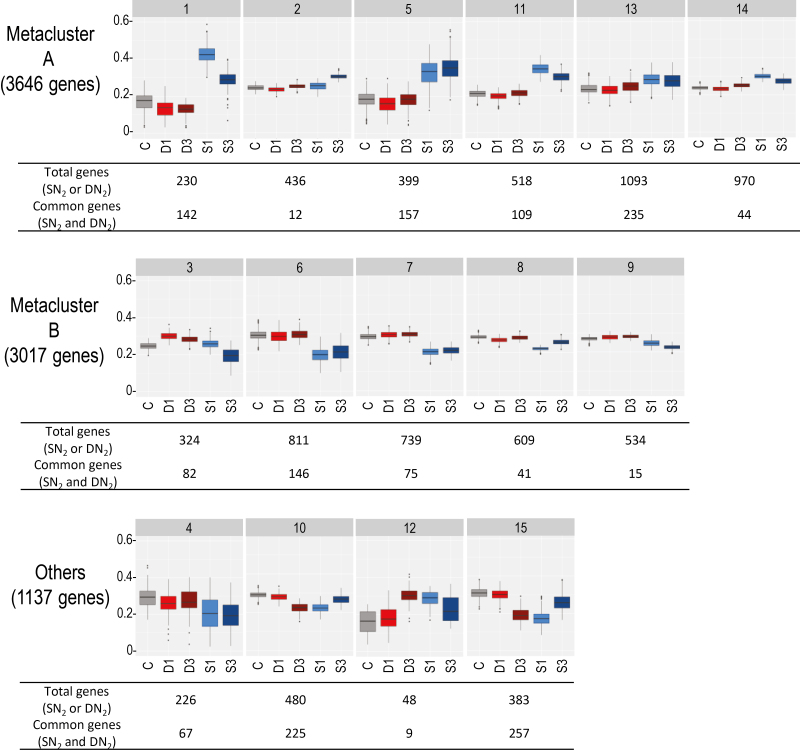
Clusters and metaclusters of DEGs co-expressed in response to systemic N signaling. Normalized expression profiles of genes (relative transcript accumulations) are represented per cluster using box plots. C, D1 and D3, and S1 and S3 correspond, respectively, to control (gray), DN_2_ (1 d and 3 d of N-deficit treatment in red and brown), and SN_2_ (1 d and 3 d of N-satiety treatments in light and dark blue), as the samples described in Fig. 1. The total number of DEGs (i.e. differentially expressed in response to N-satiety or N-deficit) as well as the numbers of common DEGs (i.e. differentially expressed in response to N-satiety and N-deficit) of the clusters are indicated.

**Fig. 6.**
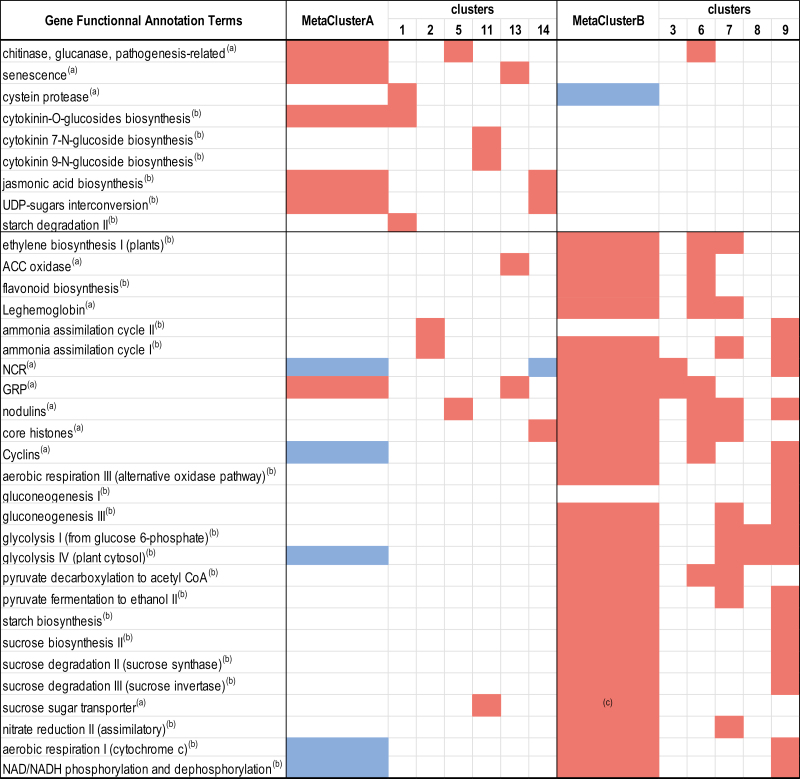
Enrichment in the functional annotation terms of the co-expressed DEGs as compared with their representation in the whole genome by hypergeometric tests. Red and blue colors correspond, respectively, to enriched and underenriched clusters or metaclusters (*P*-value <0.05). Complete data are given in Supplementary Table S11. ^(a)^Mtv4.2 gene annotation term. ^(b)^Plant Metabolic Network MedicCyc annotation term. ^(c)^*P*-value=0.058.

Co-expression analysis was performed on plant DEGs to cluster genes displaying closely related responses to systemic N signaling. The procedure was able to classify 86% of total DEGs within 15 clusters (Fig. 5; Supplementary Table S9). We combined 11 clusters (representing 85% of the total plant DEGs) in two metaclusters. Metaclusters A (clusters 1, 2, 5, 11, 13, and 14) and B (clusters 3, 6, 7, 8, and 9) were formed by regrouping clusters displaying respectively up- and down-regulation responses to N-satiety signaling. Most DEGs correspond to genes expressed in the various zones of the mature nodules described by Roux *et al.* (2014) (Supplementary Table S10). The transcript accumulations of the genes belonging to the A and B metaclusters were not equivalently spatially distributed in nodules. The A and B metacluster genes were abundant in the early infection zone, but the A metacluster genes were particularly active in the N_2_-fixing zone containing symbiosomes and differentiated bacteroids, whereas B metacluster transcripts were particularly abundant in the meristematic and the late infection zone (Supplementary Table S10). Enrichments of particular plant gene annotations were estimated by hypergeometric tests. The frequencies of annotations in clusters/metaclusters were compared with frequencies in the entire transcriptome to estimate whether observed enrichments were the result of chance or possibly related to a biological function (Fig. 6; Supplementary Tables S9, S11). Globally, DEGs were enriched in genes of the symbiotic-related islands (SRIs) physically clustered within the *M, truncatula* genome (Pecrix *et al.*, 2018), illustrating the strong impact of systemic N signaling on symbiosis (36% of the SRI genes were DEGs).

Metacluster A gene annotations were enriched with the keyword ‘senescence’. Consistently, the transcript encoding MtNAC969 (Medtr4g081870; de Zélicourt *et al.*, 2012), a transcription factor controlling nodule senescence, was found in cluster 5. Furthermore, cluster 1 was enriched for cysteine protease transcripts involved in the proteolysis of bacteroids during nodule senescence. The corresponding transcripts were down-regulated in response to N-deficit, suggesting that they were instrumental in both N responses (Supplementary Figs S3, S4). Three genes encoding cystatin, an inhibitor of cysteine protease (van Wyk *et al.*, 2014; clusters 14, 5, and 11; Medtr3g084780, Medtr4g01762, and Medtr3g043750, respectively) were also up-regulated in response to N-satiety systemic signaling. In addition, gene annotations of the metacluster A and particularly cluster 5 were strongly enriched with keywords ‘chitinase’ or ‘glucanase’ or ‘pathogenesis-related’ (16 transcripts), indicating that systemic N-satiety signaling stimulated the accumulation plant transcripts related to defense responses against microorganisms. This stimulation paralleled the up-regulation of the jasmonic acid biosynthesis genes (11 transcripts in metacluster A). Nevertheless association of these functions with the response to systemic N-satiety signaling was not systematic, as other transcripts annotated as ‘chitinase’ or ‘glucanase’ or ‘pathogenesis-related’ were enriched in down-regulated gene clusters or clusters displaying other expression profiles (clusters 6, 4, 10, 12, and 15), suggesting that transcripts related to defense against microorganisms were contributing to several N signaling responses. Metacluster A transcripts were enriched in annotations related to CK synthesis and degradation: two-component response regulators (eight transcripts), and enzymes involved in the inactivation of CKs by glycosylation (five transcripts). The up-regulation of these transcripts in response to N-satiety parallels the repression of the accumulation of three other transcripts belonging to metacluster B encoding isopentenyl transferase (IPT; clusters 6 and 7) and CK riboside 5'-monophosphate phosphoribohydrolase (cluster 12), both involved in the biosynthesis of active CKs, but also a transcript encoding CK oxidase (cluster 7) involved in the inactivation of CKs.

Genes belonging to metacluster B were repressed by systemic N-satiety signaling. The down-regulation of the transcripts encoding leghemoglobins (clusters 6 and 7; Supplementary Fig. S5), ammonium assimilation, and l-asparagine synthesis (both pathways enriched in metacluster B) paralleled the repression of SNF. The expression of many metacluster B genes marked the repression of sugar metabolism and transport in response to systemic N-satiety systemic signaling. Transcripts related to pathways of glycolysis, gluconeogenesis, sucrose, and starch metabolism were represented in metacluster B. Metacluster B also included 11 transcripts annotated as involved in sucrose transport. Striking examples were transcripts encoding the main nodule efflux sucrose transporters, MtSWEET11 (Medtr3g098930), MtSWEET15c (Medtr7g405730), and MtSWEET12 (Medtr8g096320), that were down-regulated by N-satiety. However, enrichment in sugar transporter transcripts was found in metacluster A (namely in cluster 11; six transcripts). These transcripts were up-regulated during nodule senescence and might be involved in the remobilization of sugars from the senescent organs to the plant. Whether these different behaviors of sugar transporter transcripts are related to the direction of the sugar flux mediated by these transporters remains to be determined. Although transcripts encoding enzymes involved in ethylene biosynthesis may display different responses to systemic N signaling, this annotation was globally overenriched in metacluster B (10 transcripts). Many transcripts associated with nodule formation and/or development belong to metacluster B. Repression by N-satiety signaling of 35 core histone and 18 cyclin transcripts (enriched in metacluster B; Supplementary Tables S9, S11) were probably correlated with a reduction of nodule cell division. Metacluster B was enriched in numerous nodule-specific transcripts: 24 encoding GRP peptides (Supplementary Fig. S6), 257 encoding NCR peptides (Supplementary Fig. S8), and 20 others annotated as ‘nodulins’ (Supplementary Fig. S7). Two subgroups of NCR transcripts were easily discriminated based on their accumulation kinetics in response to N-satiety systemic signaling: early (before 1 d of treatment; 114 trancripts) or late (between 1 d and 3 d treatment; 101 genes) down-regulation. In addition, several key transcripts associated with flavonoid biosynthesis (overenriched in metacluster B; nine transcripts), as well as *Rhizobium* infection and nodule formation, such as MtPUB1 (Medtr5g083030), MtDMI1 (Medtr5g083030), MtLIN (Medtr1g090320), MtRPG (Medtr1g090807), MtEFD (Medtr4g008860), MtMMPL1 (Medtr5g036083), MtVPY (Medtr5g083030), MtNFY-A1 (Medtr1g056530), MtNOOT1 (Medtr7g090020), MtDME (Medtr1g492760), MtN21 (Medtr3g012420), and MtN6 (Medtr1g062710), also belonged to metacluster B. Some of the transcripts involved in nodule development were also up-regulated by N-deficit (cluster 3); that is, most of the NCRs (including MtDNF4/NCR211; Medtr4g035705) and GRPs accumulated transcripts as well as MtDME and MtN13. Nevertheless, not all key genes of early or late nodule development are down-regulated by N-satiety signaling. For example, *MtENOD11* (Medtr3g415670), *MtNIN* (Medtr5g099060), *MtFLOT2* (Medtr3g106420), *MtSymRem* (Medtr8g097320), *MtLYK3* (Medtr5g086130), *MtRSD* (Medtr3g063220), *MtDNF2* (085800), and *MtSymCRK* (Medtr3g079850) are expressed but did not respond significantly, while *MtNSP1* (Medtr8g020840) and *MtENOD40* (Medtr8g069785) were slightly up-regulated (in metacluster A).

Finally, we paid particular attention to the 15 transcripts detected in mature nodules encoding CLE and CEP peptide hormones. A number of them were differentially accumulated in response to systemic signaling (11 CLE and two CEP transcripts; Supplementary Fig. S8). Their accumulation profiles were diverse as they are related to either metacluster A (one CEP and four CLE transcripts) or metacluster B (three CLE transcripts), or display other expression profiles (one CEP and four CLE transcripts), suggesting that they may be associated with multiple responses to systemic N signaling.

## Discussion

This study shows that the whole-plant N status triggers systemic signaling impacting mature nodule functioning and development associated with massive metabolic changes and transcriptome reprogramming(s).

The whole-plant N-satiety treatment results in the systemic repression of SNF. This down-regulation occurs within hours after providing a high level of mineral N to the treated side of the split-root system (Fig. 2) and is correlated with the systemic activation of the senescence of the N_2_-fixing bacteroids (Fig. 3). At the transcriptional level, these responses correlate with the down-regulation of the leghemoglobin gene family, several transcripts involved in ammonium assimilation, as well as in the rapid up-regulation of the transcripts encoding nodule cysteine proteases and many other plant proteins involved in nodule senescence, including the MtNAC969 transcription factor. Up-regulation of cysteine proteases and MtNAC969 transcripts during nitrate-induced nodule senescence has been previously reported (Pérez Guerra *et al.*, 2010; de Zélicourt *et al.*, 2012; Pierre *et al.*, 2014). This up-regulation does not require the presence of nitrate at the periphery of the nodules *per se*, but is under the control of the nutrient status of the whole plant. Our data are compatible with the hypothesis of nodule senescence being the cause direct of the down-regulation of N_2_ fixation, but does not rule out that additional mechanisms may also contribute to this repression. Systemic N-satiety signaling stimulates accumulation of transcripts encoding chitinase, glucanase, and pathogenesis-related proteins, as well as transcripts involved in jasmonic acid biosynthesis (van Loon *et al.*, 2006; Bari and Jones, 2009) that are known components of the pathogen-triggered immunity response generally attenuated during nodule formation (Berrabah *et al.*, 2015; Gourion *et al.*, 2015). The re-activation of defense responses against microorganisms parallels the arrest of the symbiotic association during systemically induced nodule senescence. Systemic N satiety signaling down-regulates the accumulation of numerous transcripts involved in cell division and meristematic activity (cyclin, core histones) as well as transcripts specific to the symbiotic organ development program such as transcripts encoding nodulins, GRPs, and most of the transcripts of the large NCR peptide family. Interestingly, two classes of NCR transcripts are discriminated based on their early or late responses to systemic N-satiety signaling. Diversity within the NCR gene family related to transcript accumulation kinetics, impact on bacteroid differentiation, and host specificity have already been reported (Guefrachi *et al.*, 2014; Kereszt *et al.*, 2018). Whether the differential responses to N-deficit signaling are related to the distinct functions of these peptides remains to be investigated. Previous work has evidenced that the systemic N-deficit signaling has no clear effect on nodule SNF specific activity (SNF per biomass of nodule) but stimulates nodule expansion that finally results in elevating nodule SNF in the long term (SNF per nodule; Ruffel *et al.*, 2008; Jeudy *et al.*, 2010; Laguerre *et al.*, 2012). Although the impact of N-deficit on mature nodule expansion is hardly measurable after 3 d, we observed an early inhibition of bacteroid senescence, an early down-regulation of nodule cysteine protease gene expression, as well as an early stimulation of the accumulation of NCR and GRP peptide transcripts in response to N-deficit systemic signaling. As NCRs and GRPs have been implicated in early and late bacteroid differentiation (Kereszt *et al.*, 2018), these responses may mark the early stimulation of the bacteroid differentiation that has been already characterized as a long-term response to N-deficit signaling (Laguerre *et al.*, 2012). Massive reprogramming was not observed in symbiotic bacteria as the main variations affecting bacterial transcripts were probably indirectly related to the lysis of bacteroids rather to a direct response of bacteroid gene expression to N signaling. Globally the data are consistent with the view of plant systemic N signaling having the driving role in determining mutualism behavior of the mature symbiotic organs. Altogether the data give further support to the model of bacteroid differentiation and persistence being tuned by the plant through activation of defenses and senescence (Berrabah *et al.*, 2015), and further suggest that systemic N signaling pilots these controls. However, at this stage, it cannot be ruled out that variation of genetic expression in bacteroids operates at the post-transcriptional level. A striking example that may argue for post-transcriptional regulation in bacteroids in response to N signaling is the apparent discrepancy between the impact of N-deficit on the ‘GABA shunt’ activity in mature nodules that is well observed at the level of metabolite accumulation but not associated with clear variation of the accumulation of the transcripts encoding the related enzymes.

Systemic N signaling triggers major variations of the metabolite pools of the mature nodule that are inversely correlated with the whole-plant N demand. They illustrate the variations of the exchanges between nodules and the whole plant. Whole-plant N-satiety and N-deficit signaling, respectively, trigger a dramatic reduction and substantial increase of sugar pools within the first day of treatment. These variations are correlated with changes of the levels of transcripts involved in glycolysis, sucrose metabolism, starch degradation, and biosynthesis. Because most of these responses tend to compensate sugar pool variations, they are likely to be the consequences rather than the cause of changes of the sugar content of the nodule. As the sucrose produced by photosynthesis and translocated from the shoot to the root through the phloem stream is the primary source of nodule carbon metabolites (Udvardi and Poole, 2013; Liu *et al.*, 2018), the sugar pool variations are probably due to changes in the phloem-driven sucrose partitioning from the shoot to the symbiotic organ rather than to nodule metabolism activity. Interestingly, variations in the sugar content of the nodule correlate with variations of the accumulation of several transcripts involved in sugar transport, some encoding SWEET sugar transporters that are potentially involved in the sugar export from the source tissue to the nodule (Kryvoruchko *et al.*, 2016). Variations of assimilate partitioning from the shoot to the nodules have already been correlated with early systemic N-deficit signaling using ^13^CO_2_ labeling in *M. truncatula* nodulated plants exposed to a local suppression of N_2_ fixation (4 d of Ar/O_2_ treatment; Jeudy *et al.*, 2010). These N-deficit systemic responses include rapid variation of sugar content of the nodule already measured after 1 d of partial arrest of SNF. N-satiety is associated with the reduction of the asparagine and glutamate pools produced by the assimilation of the NH_4_^+^ by SNF, as well as the malate pool, the primary carbon and energy source provided by the plant to the bacteroid (Udvardi and Poole, 2013). N-satiety also reduces the GABA, alanine, and succinic semialdehyde pools that mark the activity of the so-called ‘GABA shunt’. This metabolic pathway was suggested to allow the hypoxic symbiotic tissues to maintain the efficiency of the TCA cycle (Prell *et al.*, 2009) and was proposed to be regulated by the plant through GABA partitioning from the shoot to the nodule (Sulieman and Schulze, 2010; Sulieman, 2011). N-deficit signaling mirrors N-satiety systemic responses by increasing these metabolite pools. Amino acids have been suggested as potential signals for systemic N regulation of symbiosis, indicating a specific ‘N-feedback’ mechanism (Parsons *et al.*, 1993; Bacanamwo and Harper, 1997; Sulieman and Schulze, 2010; Sulieman *et al.*, 2010). Nevertheless, although the levels of many amino acids and some organic acids vary in response to systemic N signaling, their accumulation kinetics are late as compared with sugars, early transcriptome reprogramming, and SNF (in the case of N-satiety). Therefore, the variatons in amino acids and organic acids are more likely to be consequences of sucrose partitioning. Sugar allocation by the plant to the nodule may behave as a systemic metabolic signal that drives nodule function and development. Earlier reports indicated that (i) carbon partitioning is limiting nodule development and function at vegetative stages (Voisin *et al.*, 2003); and (ii) increasing partitioning of photosynthates from the shoot to the roots by elevating ambient CO_2_ enhances SNF in legumes (Rogers *et al.*, 2006). Furthermore the role of SWEET transporters in the control of the plant interaction with pathogenic microbes has already been documented (Bezrutczyk *et al.*, 2018). This work suggests that these transporters may also contribute to adjusting the benefits of beneficial microorganisms to plant nutritional demand. However, how the whole-plant N status is perceived in order to modulate sucrose allocation remains to be discovered.

Although the variation of sugar allocation is, to our knowledge, the earliest known response to systemic N signaling in mature nodules, it cannot be excluded that there is a consequence of reprogramming of nodule development by hormonal and/or peptide signals. Systemic N signaling modulates the accumulation of a number of transcripts involved in (i) the CK transduction pathway as well as in CK inactivation and biosynthesis; (ii) ethylene biosynthesis; (iii) jasmonic acid biosynthesis; and (iv) expression of several genes encoding CEP and CLE peptide hormones. All of these hormones and peptides can be mobile signals, have been implicated in the control of nodule formation and development (Ferguson and Mathesius, 2014; Buhian and Bensmihen, 2018), and therefore could be involved in the systemic control of mature nodules. Further investigations are required to discriminate whether these hormones and peptides may be direct systemic signals of N status of the plant or secondary components of the systemic N responses.

This study illustrates that the function and development of the symbiotic organs are highly integrated at the whole-holobiont level. Systemic regulations could be interpreted according to a mutualism model based on plant C–N trade-offs. Plants under N-deficit stimulate SNF by allocating photosynthates toward N_2_-fixing mature nodules, whereas when the N demand is satisfied by addition of mineral N the plants shut down this allocation and promote symbiotic organ senescence as the C cost of SNF is excessive as compared with mineral N nutrition. Because of climate change, atmospheric ambient CO_2_ is expected to increase, modifying the conditions of symbiotic C–N trade-offs (Rogers *et al.*, 2006). How this may consequently modify the equilibrium of *Rhizobium*–legume mutualism is an open question that deserves further investigation.

## Supplementary data

Supplementary data are available at *JXB* online.

Fig. S1. Global view of responses of the mature nodule transcriptome to N signaling.

Fig. S2. Global comparison of the plant transcriptome responses identified in this study with the previous data of Ruffel *et al.* (2008) and Cabeza *et al.* (2014).

Fig. S3. Heat map of transcript accumulation of plant DEGs annotated as cysteine proteases.

Fig. S4. Heat map of transcript accumulation of expressed plant genes annotated as proteases.

Fig. S5. Heat map of transcript accumulation of plant DEGs annotated as plant hemoglobins.

Fig. S6. Heat map of transcript accumulation of plant DEGs annotated as glycine-rich peptides (GRPs).

Fig. S7. Heat map of transcript accumulation of plant DEGs annotated as nodule cysteine-rich (NCR) peptides.

Fig. S8. Heat map of transcript accumulation of expressed plant genes annotated as CLE or CEP peptides.

Fig. S9. Box-plot representation of the relative accumulation of leghemoglobin, cysteine protease, and sweet 11 transcripts in response to systemic N-satiety or N-deficit signaling in three independent split-root experiments.

Table S1. Effect of systemic N signaling on sugar contents of mature nodules.

Table S2. Effect of systemic N signaling on soluble amino acid contents of mature nodules.

Table S3. Effect of systemic N signaling on organic acid contents of mature nodules.

Table S4. RNAseq data of the plant genes expressed in control, DN_2_, and SN_2_ mature nodules (log_2_-normalized counts).

Table S5. Differential expression of N-deficit and N-satiety plant DEG transcripts in the various temporal contrasts of the analysis (DE_groups).

Table S6. Gene Ontology (GO) enrichment comparison between N-satiety and N-deficit plant DEGs.

Table S7. RNAseq data of the bacterial genes expressed in control, DN_2_, and SN_2_ mature nodules (log_2_-normalized counts).

Table S8. Differential expression of N-deficit and N-satiety bacterial DEG transcripts in the various temporal contrasts of the analysis (DE_groups).

Table S9. Assignment of total plant DEGs to co-expression clusters and metaclusters.

Table S10. Preferential expression of the DEGs in the nodule zones deduced from the data of Roux *et al*, 2014.

Table S11. Enrichment in the functional annotation terms of the co-expressed DEGs as compared with their representation in the whole genome (Mtv4.2) by hypergeometric tests.

eraa221_suppl_Supplementary_Table_S1

eraa221_suppl_Supplementary_Table_S2

eraa221_suppl_Supplementary_Table_S3

eraa221_suppl_Supplementary_Table_S4

eraa221_suppl_Supplementary_Table_S5

eraa221_suppl_Supplementary_Table_S6

eraa221_suppl_Supplementary_Table_S7

eraa221_suppl_Supplementary_Table_S8

eraa221_suppl_Supplementary_Table_S9

eraa221_suppl_Supplementary_Table_S10

eraa221_suppl_Supplementary_Table_S11

eraa221_suppl_Supplementary_Figure_S1_S9
