# Supplementary material for: Responses of mature symbiotic nodules to the whole-plant systemic nitrogen signaling
Source: J Exp Bot. 2020 May 9;71(16):5039–52. doi: 10.1093/jxb/eraa221 (PMC7410188; doi:10.1093/jxb/eraa221)
Supplement: eraa221_suppl_Supplementary_Figure_S1_S9 [file eraa221_suppl_supplementary_figure_s1_s9.pdf]

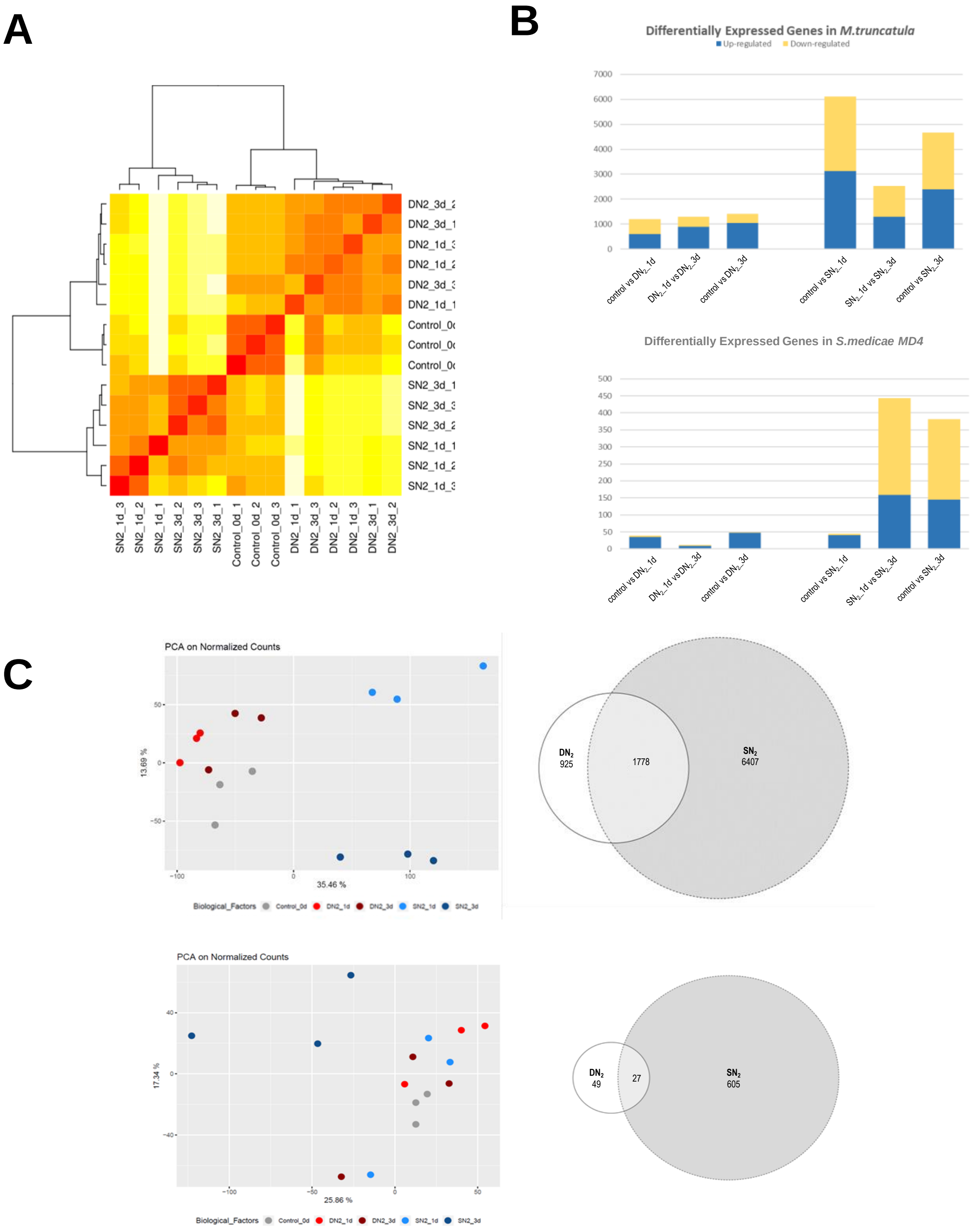

Supplemental Figure S1. **Global view of responses of the mature nodule transcriptome to N-signaling.** Sample names are designed as following: N-treatment_duration of the treatment_repeat number. Panel A represents the hierarchical clustering of RNAseq data at the whole transcriptome level (normalized numbers of reads). As expected SN_2, C and DN_2 samples cluster separately. Panel B contains histograms of plant and bacterial DEGs according the analyzed contrast. Panel C represents the principal component analysis (PCA) of the RNAseq data per samples and the Venn diagrams of N-satiety and N-satiety DEGs. Upper panel C correspond to plant DEGs and lower panel C to bacterial DEGs. The PCA analysis revealed that two major components are discriminating efficiently plant RNAseq data according the treatment and the duration of the treatment. However bacterial RNAseq data were poorly discriminated by PCA analysis except for 3 days N-satiety treatment.

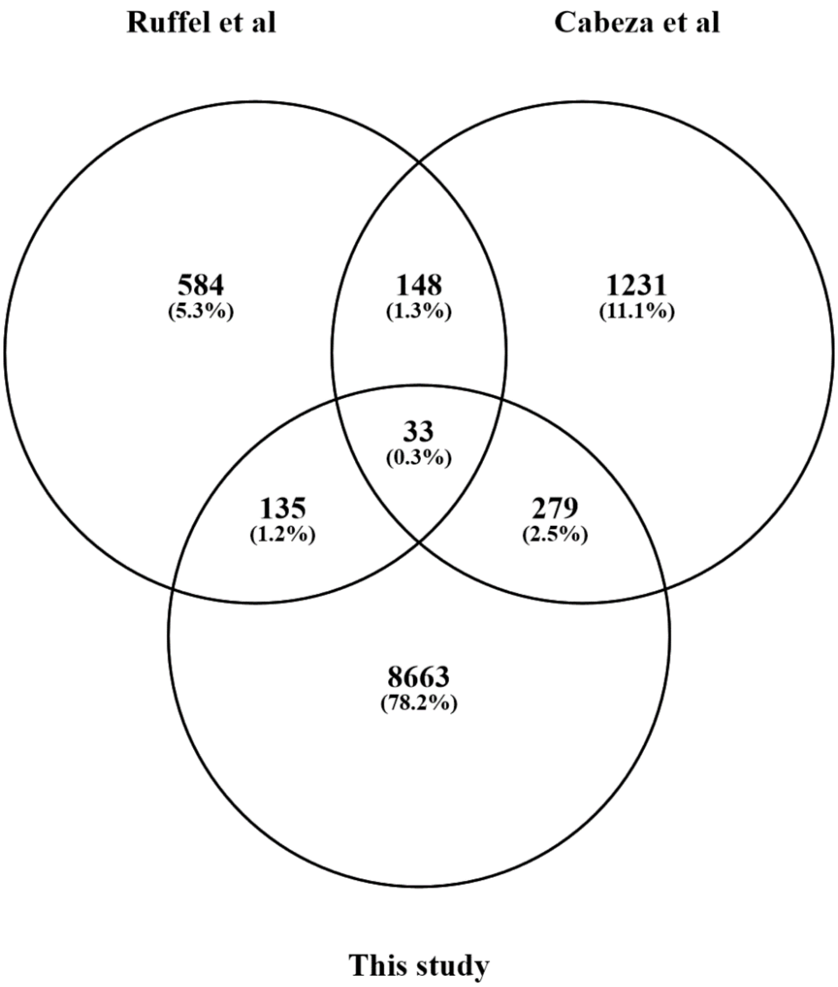

Supplemental Figure S2. Global comparison of the plant transcriptome responses identified in this study to the previous data of Ruffel et al . (2008) and Cabeza et al. (2014). As estimated by pairwise hypergeometric tests, the overlaps between the three datasets were not the result of chance ($p<0,05$). However these overlaps remain relatively limited. These two studies described the systemic responses of the nodulated root to the whole plant N demand and the local responses of nodules to nitrate, thus correspond to biological conditions and/or scales that are related but different from those of the present study.

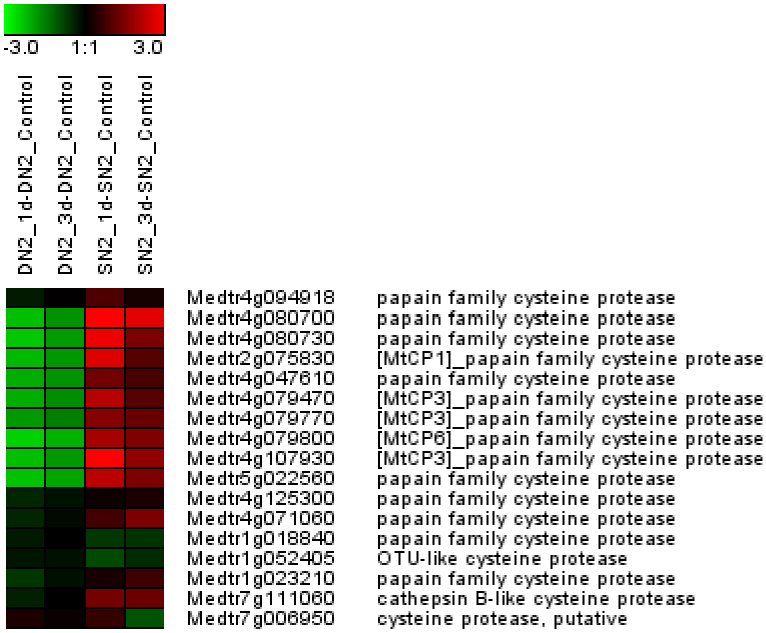

Supplemental Figure S3. Heat map of transcript accumulation of plant DEGs annotated as cysteine proteases.

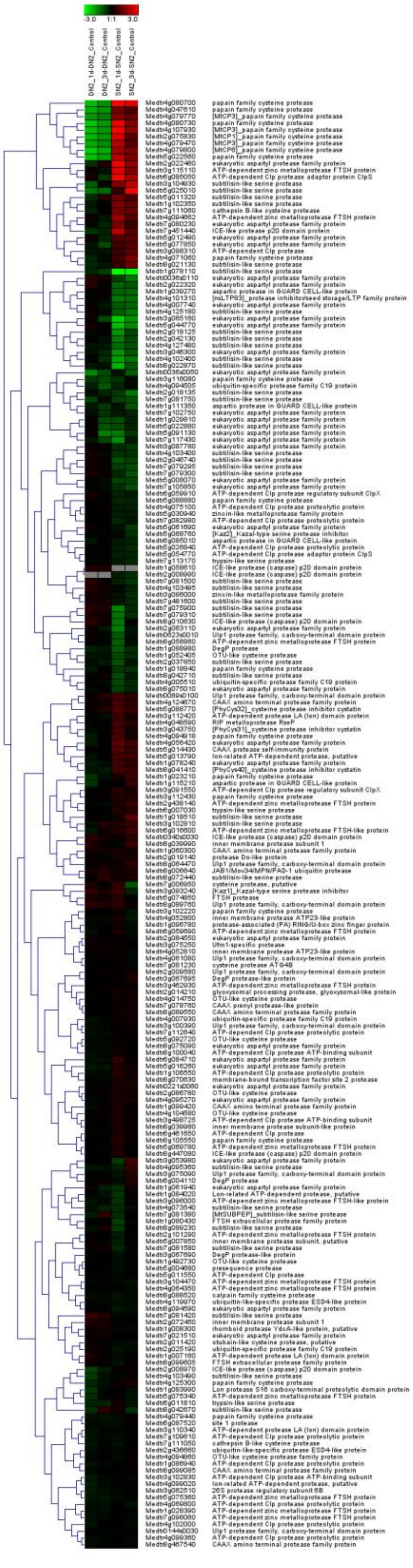

Supplemental Figure S4. Heat map of transcript accumulation of expressed plant genes annotated as proteases**.**

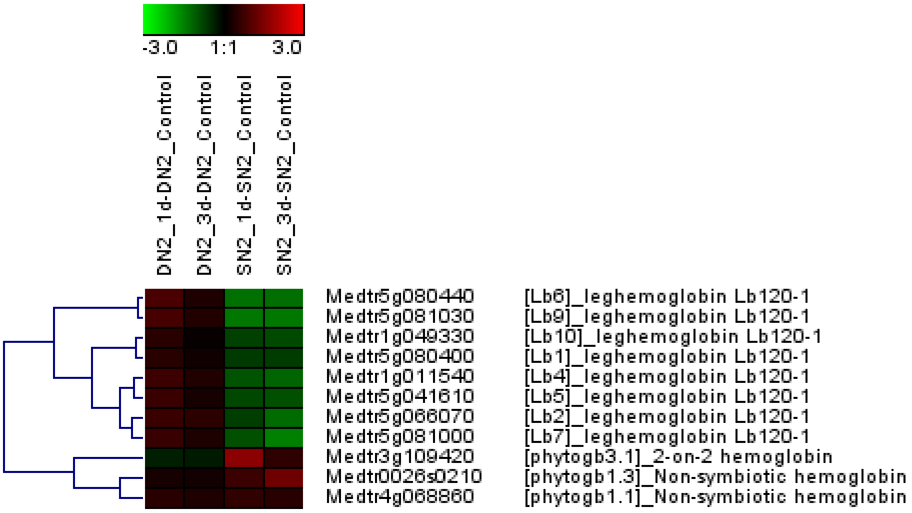

Supplemental Figure S5. Heat map of transcript accumulation of plant DEGs annotated as plant hemoglobins.

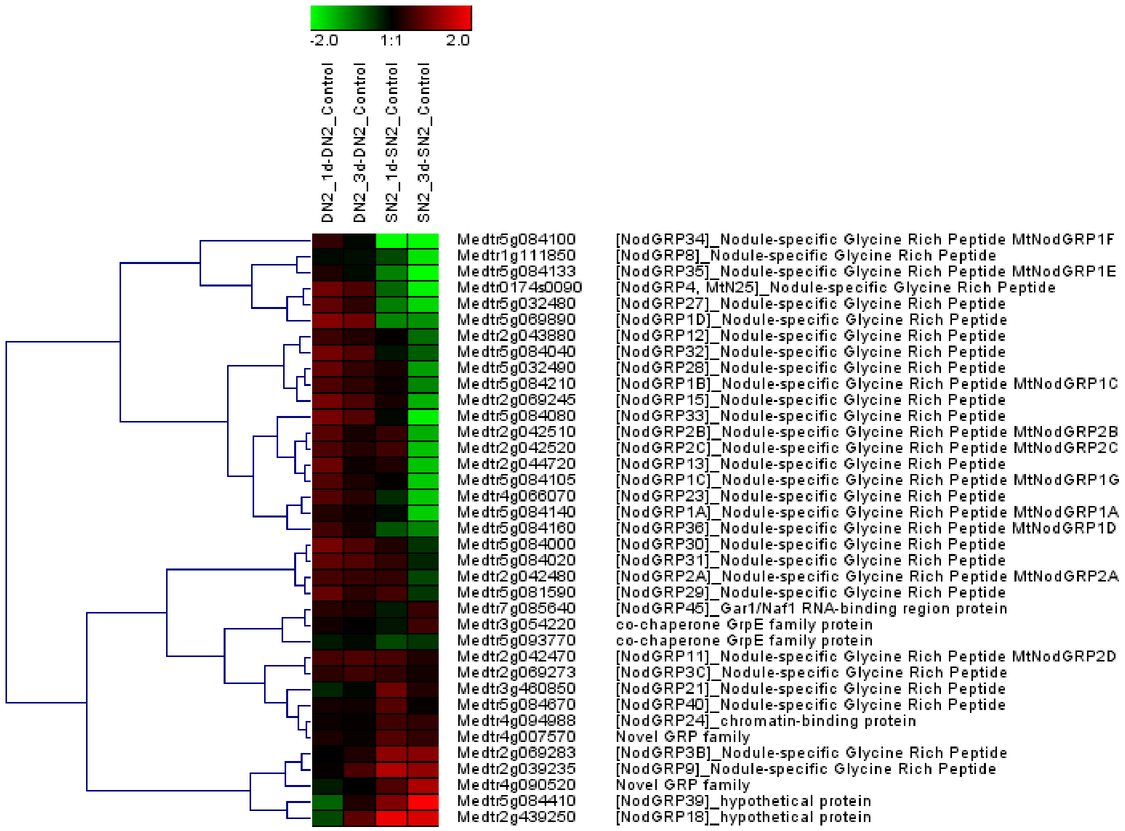

Supplemental Figure S6. Heat map of transcript accumulation of plant DEGs annotated as Glycine Rich Peptides (GRP).

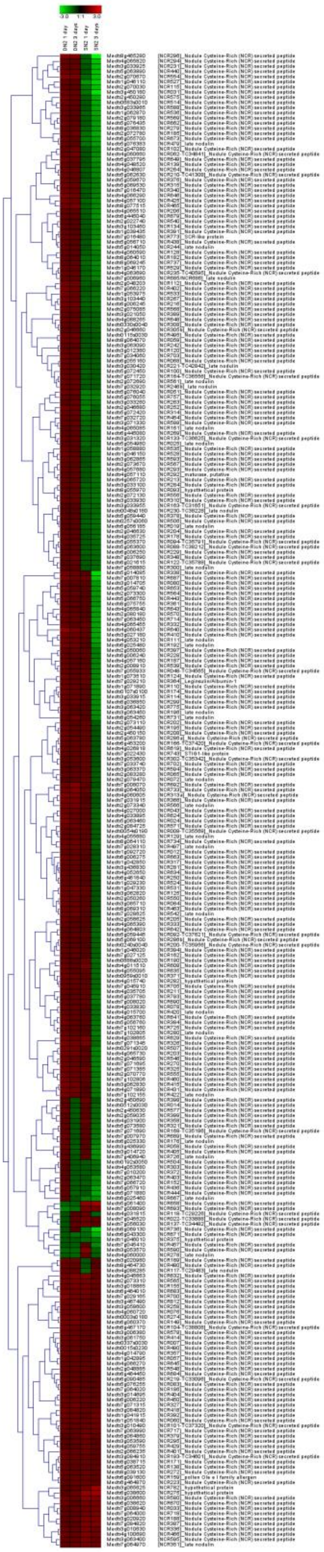

Supplemental Figure S7. Heat map of transcript accumulation of plant DEGs annotated as Nodule Cysteine Rich peptides (NCR).

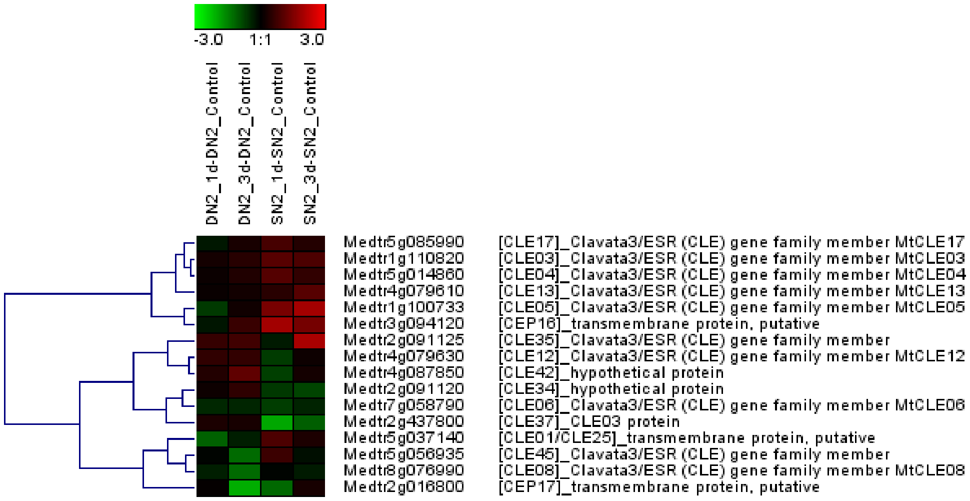

Supplemental Figure S8. Heat map of transcript accumulation of expressed plant genes annotated as CLE or CEP peptides.

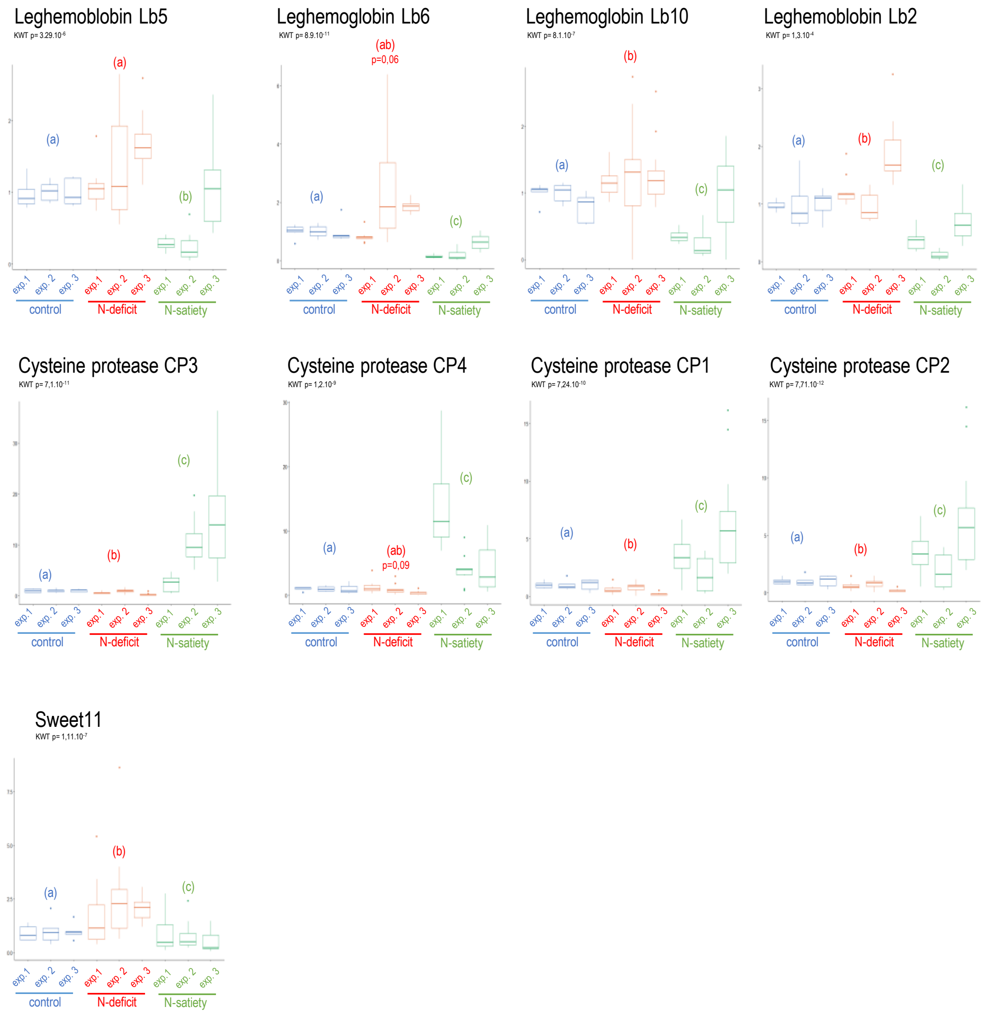

Supplemental Figure S9. Box-plot representation of the relative accumulation of leghemoglobin, cysteine protease and sweet 11 transcripts in response to systemic N-satiety or N-deficit signaling in three independent split-root experiments. . Nodules were collected from plants grown in the experimental system described in fig.1. RT-q-pCR have been done on total RNAs extracted from the root nodules of 4-6 plants (collected separately) in 3 independent split root experiments. RT-q-PCR experiments were done according Girin et al. (2007) using ACTIN11 and GAPDH transcripts as internal standard (Ruffel et al. 2008; Plet et al. 2011). In experiment 1, N-satiety and N-deficit treatments were applied for 4 and 7 days, in experiments 2 they were applied for 3 and 5 days, in experiment 3 they were applied for 1, 2 and 3 days. Effects on transcript accumulation were analyzed by Kruskall wallis test (p-value threshold <0,05) followed by pairwised Willcoxon test (adjusted p-value threshold <0,05). Letter indicates the different classes of relative expression values deduced from this test. Although quantitative variations between experiments may be occasionally observed, statistical analysis confirmed the robustness of the responses to N-deficit and N-satiety signaling of all transcripts.
